# Clotho: addressing the scalability of forward time population genetic simulation

**DOI:** 10.1186/s12859-015-0631-z

**Published:** 2015-06-10

**Authors:** Patrick P. Putnam, Philip A. Wilsey, Ge Zhang

**Affiliations:** 1 Human Genetics, Cincinnati Children’s Hospital Medical Center, 3333 Burnet Ave, Cincinnati, 45229–3026 OH USA; 2 Department of Electrical Engineering and Computing Systems, University of Cincinnati, PO Box 210030, Cincinnati, 45221–0030 OH USA

**Keywords:** Population genetic simulation, Data structures, Sequence representation, Scalability

## Abstract

**Background:**

Forward Time Population Genetic Simulations offer a flexible framework for modeling the various evolutionary processes occurring in nature. Often this model expressibility is countered by an increased memory usage or computational overhead. With the complexity of simulation scenarios continuing to increase, addressing the scalability of the underlying simulation framework is a growing consideration.

**Results:**

We propose a general method for representing *in silico* genetic sequences using implicit data structures. We provide a generalized implementation as a C++ template library called Clotho. We compare the performance and scalability of our approach with those taken in other simulation frameworks, namely: FWDPP and simuPOP.

**Conclusions:**

We show that this technique offers a 4x reduction in memory utilization. Additionally, with larger scale simulation scenarios we are able to offer a speedup of 6x - 46x.

## Background

Forward Time Population Genetic Simulations (FTPGS) are essential tools that aid in the study of complex interactions which contribute to the evolutionary process. They enable the more efficient study of allele frequency change over time as a result of a set of models that reflect naturally occurring processes such as mutation, recombination, selection, gene flow, and genetic drift.

Over the years, a plethora of Forward Time Population Genetic Simulators have been developed [1]. It is not uncommon to find simulators that perform efficiently for a very specific subset of scenarios in a given domain but fail to provide a broad solution suitable for general use [2, 3]. Several general simulation frameworks [4-6] have been developed to allow users to build their own simulator capable of addressing the scenarios they are interested in. Often, these frameworks lack support for scalable performance to study the larger simulation scenarios which many investigators are pursuing [7].

Scalability refers to the ability of a program to handle an increased amount of work. In software, this is measured in terms of both computational runtime, and resource utilization. The scalability of a simulation depends upon many elements. At a high level, a simulation is dependent upon the choice of models, configurations of those models, and the desired scope of the simulation. Fundamental to all of these is the implementation. For example, if the models are not implemented with scalability in mind, then the scalability of the entire simulation suffers.

The design and implementation of a model is often a challenging problem with potentially many dependencies interacting in various ways. For example, in FTPGS most models being explored depend upon a genetic sequence. As a result, if the representation of a genetic sequence is not scalable, then the entire simulation becomes less scalable.

### Impact of genetic sequence representation

We refer to a genetic sequence as the *in silico* representation of the genetic material specific to each individual in a population. The aim of a simulation is to, in effect, evolve a set of genetic sequences. The various models that are evaluated during a simulation may either work to modify a genetic sequence, or analyze the set of genetic sequences to identify specific characteristics. As genetic sequences are such an integral component of any FTPGS, their *in silico* representation plays a significant role in the overall scalability of a simulation.

Most FTPGS are constructed considering a genetic sequence as a locus ordered list of alleles. This design is intuitive as it mirrors that of genetic structures in nature. In general, this common data structure is easily implemented and provides relatively straightforward use. Also, the models can take advantage of the ordering to improve their efficiency. Although most simulators are built using this common structure, they often differ in their computational abstraction of an allele and the subsequent computational optimizations that may result.

An allele is generally abstracted as a symbol reflecting a specific state of a locus. From an implementation perspective, there is a choice of how the state should be represented. In some cases, it suffices to set a upper limit on the number of states for every locus. Thus, every locus can be represented as a fixed-length *value*, or symbol. For example, it may suffice to consider any site as existing in one of two states: a reference state, or a mutated state. Symbolically, these could be represented by *0* and *1*, respectively.

Simulation environments such as simuPOP [5] and NEMO [4] offer sequence representations that use a fixed-length value for each locus being simulated. In effect, this is a string representation of a genetic sequence. There are several advantages of this approach. For instance, determining the allelic state of a known locus is a constant time operation. Furthermore, some operations can be applied to adjacently ordered loci simultaneously, resulting in a reduction in computational cost. A disadvantage of the string representation is that interpretation of the symbol depends upon its location within the sequence. Therefore, all loci must be represented in every sequence, and each sequence is effectively a fixed-length string. This can result in an under utilization of memory when alternate alleles occur infrequently within a population.

One way to improve the memory utilization of a genetic sequence is to remove those loci that are in a reference state. However, simply removing such loci could potentially change the interpretation of a symbol in the sequence as the relative positioning would change. Therefore, it is also necessary to change the definition of a symbol to encode the locus specific information in addition to a state. In effect, the symbol becomes a *key*, rather than a value. The resulting genetic sequence is an indirect list.

FWDPP [6] is an example of a C++ template library that adopts this approach. By representing only keys for those loci in a non-reference state, the length of a sequence can be significantly reduced. This improves memory utilization and computational performance in some algorithms. Also, by adding a layer of indirection between the genetic sequence and its state we gain the ability to more efficiently add alleles to the population. That is, a new allele can be added to the population and only the sequence in which it appears has to be updated. A less obvious benefit is that modifying the value associated with a key can also be performed and every sequence containing that key will automatically be updated. Our proposed solution capitalizes on these benefits.

Although offering several advantages, there are some disadvantages associated with the indirect sequence representation. There are scenarios where this representation will consume more memory than an equivalent fixed-length string. In addition, it is necessary to decode, or dereference, a key to determine the corresponding locus and state. This can become a significant amount of overhead over time. We will discuss these in greater detail in later sections.

### Motivating our approach

Our design is motivated by several observations. First, although the set of alleles may be considered infinite over time, only a finite subset of these alleles will appear in any generation of a finite population. In other words, the set of alleles dynamically changes between generations. From a data structure perspective, the indirect sequence allows changes to the set of alleles to be propagated through the population more efficiently. However, during the analytic stages of a simulation the set of alleles is effectively static, or fixed. As a result, the fixed-length string representation offers several algorithmic benefits which make it more desirable.

In this work, we will describe a genetic sequence representation that combines the advantages from both fixed-length string and indirect sequences. In addition, we describe the model modifications that take advantage of the representation. We will discuss the advantages and disadvantages of this representation as they relate to fixed-length and indirect sequences, and scalability. Finally, we introduce a C++ template library called Clotho that offers a general implementation of the proposed representation.

## Methods

Our design aims to succinctly represent the genetic sequences within a generation of a population. We strive to represent only those alleles that are present in the generation, and rely upon external structures to retain information pertaining to those alleles that were at one time present in the population. Rather than returning space freed by the removal of alleles to the system, we re-purpose it to represent a new allele that arises in a future generation.

To achieve these aims we utilize an indirection technique similar to that mentioned earlier. Alleles are maintained in an indexed structure. The index of the allele effectively becomes the key of the allele. However, in our design the genetic sequence does not explicitly store the index of only those alleles which it contains. A genetic sequence is instead represented by a binary sequence that enumerates the set of alleles.

Here, we briefly review how to represent a set of elements as a binary sequence. Next, we describe how the set of alleles changes between generations and how the binary representation aids the process of identifying the changes. Then we show how a model can take advantage of this sequence representation. Finally, we present an example of the flexibility in our design.

### Sets as binary sequences

Forward Time Population Genetic Simulations are used to study the change in allele frequency over time for a population of individuals under various models. We refer to an allele as being a 2-tuple (*Π*,*Σ*), where *Π* is the genetic position, and *Σ* is the allelic state, or genetic sequence, at that position. In general, the genetic position is a right-open interval over the natural numbers, and represents the relative start and end positions of the genetic sequence. For simplicity, however, we will consider alleles as being the alternate forms for specific sites. Furthermore, we will assume that two alleles are equivalent if they represent the same genetic position and the same genetic state.

In an abstract sense, a genetic sequence is a set of non-overlapping alleles. By combining the set of alleles for each sequence in a population, a superset, *S*, can be constructed. From the opposite perspective, if the set of alleles, *S*, is known for a population, then every sequence of the population is a subset of *S*. In other words, a population is a family of sets over *S*, which we will represent as **F**.

Representing a subset of *S* as a bit sequence of length *M*=|*S*| is relatively straight forward. Each element of *S* can be assigned a specific bit index in the sequence. A set bit indicates that the element assigned to that bit is present in the subset. In general, this is how one could enumerate all possible subsets of *S*.

There are several observations to be made about this representation. First, assigning elements to bits is arbitrary as long as it remains consistent for all subsets. We will assume that there is an invertible function *f* which maps elements, in this case alleles, to bit indices, and vice versa. Second, adding new alleles to *S* does not require changing any existing subsets of *S*. That is, any sequence which exists in **F** can remain unchanged when a new allele, *A*, is added to *S*. To see this consider that new alleles may always be assigned to the next highest available bit index. Because *A* is new, existing sequences of **F** do not contain *A*. Updating existing sequences to reflect the addition of *A* amounts to appending a zero to an end of each sequence.

Generally, zero extending a sequence is not technically difficult to achieve, however it can be costly to perform. As a result it is often beneficial to consider each sequence as having a soft- and a hard-end. The soft-end is the highest set bit position in the sequence, and the hard-end is the total number of possible alleles, *M*. By definition, all bits at positions greater than the soft-end are zero, so appending more zeros becomes meaningless. However, it is useful, from a computational perspective, to pad the soft-end with some zeros to provide for better memory alignment.

**Fig. 1:**
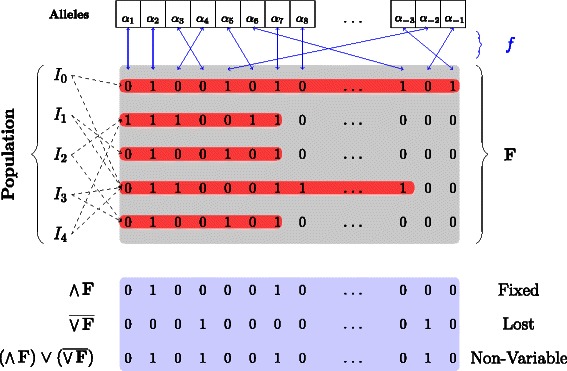
General *in silico* sequence representation overview. The unique alleles of the population are referenced by a central, indexed structure. The individuals *I*_∗_ of the population reference bit sequences in the family **F** indicated by the dashed lines. The red boxes highlight the bit sequence up to its soft-end. The blue arrows indicate the mapping between an Alleles and bit index as assigned by *f*. Metadata bit sequences representing the Fixed, Lost, and Non-Variable alleles are shown in the blue box

Utilizing this observation allows zero extension procedures to be performed on an as needed, or per sequence, basis. In effect, zero extension is only necessary when an existing sequence gains an allele that has been assigned to a bit position after its soft-end. It is worth pointing out that although each sequence may be physically represented as a variable length bit sequence, all sequences may be logically interpreted as being a fixed-length string of *M* bits. Figure 1 provides a general overview of this representation.

Enumerating the subsets of *S* in this manner also offers several computational advantages. First, the standard set operations directly translate to Boolean Algebra operations. In addition, Boolean operations can be applied to multiple bits, or blocks, in a single step as all bits are independent. Furthermore, the performance of a set operation is no longer dependent on which elements are present in the set, rather they depend upon the maximum number of elements in *S*. That is, the Boolean operations are dependent upon the length of sequence and the width of a block, and not the states of the bits in the block. In general, the performance of Boolean operations on a bit sequence is $O(\frac {M}{W})$, where *W* is the width of a block in bits. Often, *W* is the word size of a processor.

The representation of genetic sequence as a bit sequence is not a new concept. Indeed, bit sequences have been used to represent sets of known bi-allelic loci [5], or at points in time during a simulation when the set of alleles is known [8]. We intend to show that they may also be used when *S* is dynamic.

### The alleles of a population

The population’s set of alleles, *S*, changes with each generation as a result of natural processes such as mutation and fixation. These processes act to either create new, or remove alleles within a population. Over time we expect some alleles will become *lost* or *fixed* in a population. An allele is considered *lost* if the allele does not exist in any sequence of the current generation. Conversely, a *fixed* allele is present in every sequence of the population. We term alleles that are neither fixed nor lost as being *variable*.

As mentioned earlier, our representation strives to represent only the variable alleles within a generation. Variable alleles are of most interest in Population Genetics as they represent the alleles which make individuals unique. Non-variable alleles may provide valuable information, however, from a computational perspective, representing non-variable alleles in every sequence of a population amounts to an under utilization of computational resources. For example, if we knew *a priori* that allele *A* is fixed in the population, then by definition every sequence of the population has *A*. In effect, the representation of *A* in every sequence amounts to redundant information. That is, if we could externally preserve the fact that *A* is fixed in the population, then we could effectively remove *A* from every sequence without losing any information about the population. This would reduce the amount of physical memory used to represent each sequence. Furthermore, any algorithm which operates over the length of sequence would perform more quickly since the sequence length has been reduced. Therefore, the identification of non-variable alleles is an important component to achieving a scalable simulation.

Identifying the non-variable alleles within a population amounts to performing basic set theoretic operations on the population’s sequences. The fixed alleles are easily found by performing a set intersection of the sequences within the current generation of a population. Lost alleles are a bit trickier. In order for an allele to be lost, it had to have existed at one time. This means that a lost allele exists in the set of possible alleles *S*, but does not exist in any of the sequences in the most recent generation. In terms of set operations, this set is determined by taking the set difference of *S* and the union of the population’s sequences.

The bit sequence representation makes identification of non-variable alleles a straightforward and efficient procedure. Computing the intersection of the bit sequences is equivalent to computing the bit sequence which is the bitwise AND of all the bit sequences of **F**. Similarly, the set of lost alleles is the set of bit positions which are all unset in every sequence. More specifically, it is the bitwise negation of the bitwise OR of all sequences of **F**. We term the union of the fixed and lost sets as being the set of *free* alleles. It is the bitwise OR of the fixed and lost bit sequences. Figure 1 also illustrates these sets in the metadata region.

The *free* bit positions amount to unused memory in **F**. Ideally, for a scalable solution we strive to minimize the amount of unused memory. However, here we opt to allow it to remain under the assumption that it will be reused in the future by new alleles. This allows us to simply copy a sequence from a parent generation to a child generation, operating upon the child sequence as necessary. This is advantageous because we avoid performing a costly bit shifting algorithm on sequences between generations.

New alleles resulting from the natural processes can be added to *S* following a simple algorithm. First, replace an allele associated with a *free* bit position, and remove the bit position from the *free* set. If the *free* set is empty, then the new allele can simply be appended to the bit sequence. It is worth noting that replacing an allele associated with a *free* bit position does not impact already existing sequences in **F**. By definition, no sequences in **F** contain an allele currently occupying a *free* bit position. Therefore, replacement will not result in any loss of information.

### Algorithmic impact

Algorithms that depend upon the existence of an allele rather than the value of an allele can best leverage the bit sequence representation. However, many algorithms in FTPGS do depend upon the properties of an allele. These algorithms generally incur an additional computational overhead when the bit sequence representation is used. The majority of the computational overhead is a result of having to perform bit walking to locate desired bit states, and subsequently dereference the value. Here we focus on minimizing the cases where bit walking is required using a simple recombination algorithm as an example.

Recombination is a naturally occurring process by which two parental chromosomes exchange genetic material generally resulting in a new child chromosome. This process is modeled as a copy and conditional swapping of two sequences. From an algorithmic perspective, the process begins with two parental sequences, *P*_0_ and *P*_1_. The child sequence, *C*_0_, is constructed by iteratively copying one parental sequence until a crossover event is encountered, which causes the swap of the source parental sequence.

The algorithm for this process can be broken into two steps: generation of crossover events, and construction of the child sequence. Generating a list of crossover events generally allows for more efficient block copying routines to be used during the child sequence construction. As an example, if the genetic sequences are represented as fixed-length locus ordered strings, then the events serve as sequence offsets. Computationally this means we can perform a direct memory copy between offsets of the parent sequence to the appropriate child sequence. This is generally more efficient than copying sequences locus by locus.

With our genetic sequence representation the general steps of recombination remain the same, however the construction of a child sequence takes a slightly more general form as the alleles are not necessarily naturally ordered. First, notice that the crossover events effectively define a set of chromosomal segments. These segments can be categorized as being *maternal* or *paternal* segments. That is, the bit state of *C*_0_ for all alleles within paternal segments will be copied from *P*_0_; the bit state of *C*_0_ for all alleles residing within maternal segments will be copied from *P*_1_. A basic algorithm for constructing a child sequence would iterate over the set of alleles, classify each allele according to the segment that they reside within, and copy the state of the allele from the appropriate parent.

While the above procedure serves as the basic algorithm, as stated it is a less than desirable process. Not only does it involve classifying every allele, but it also involves bit walking multiple sequences. Ideally classification should be limited to only those alleles for which the parents differ. That is, if both parental sequences share a state for a specific bit position, then the parental source of the allele is technically not important as the child sequence will inherit that state. Identification of shared or different bit states in a block is done by computing the intersection or symmetric difference, respectively.

The resulting algorithm is to build the child sequences a block of *W* bits at a time. For each pair of blocks from the parent sequences, a block, *D*, representing the symmetric difference, or bitwise XOR, of the pair is computed. If *D* contains at least one differing bit, then it is necessary to bit walk *D* and classify each of the corresponding allele according to the set of chromosomal regions. The classification step results in a block of bits where set bits indicate alleles residing within maternal segments. This bit block is used to mask the bits from parental sequences to construct the child blocks accordingly. Finally, the child blocks are appended to their respective sequences. The general algorithm is provided in Algorithm 1.



While this symmetric difference observation helps to reduce the amount of bit walking, classifying each differing allele may still result in a significant amount of overhead. A further reduction may be achieved if more information about the alleles can be discerned from the bit sequence. That is, it is possible to utilize the ordering of alleles at the bit sequence level.

### Ordering the alleles of a population

To this point, we have made minimal use of the allele ordering resulting from the allele indexing function *f*. We have relied upon the constraint that the mapping of an allele to a bit position is defined at the population level, and simply assumed that each allele is ordered independently of one another. Indeed, the order of alleles does not have a direct impact on the performance of set operations. However, other algorithms may be able to leverage a relative ordering to achieve better scalability.

Consider the recombination algorithm mentioned earlier. For genetic sequences that are fixed-length strings of well-ordered loci, the copy and conditional swapping can take advantage of block copying. As the bit sequence representation is effectively a fixed-length string, if we can order the alleles of the population by their genetic location, then we should be able to adapt this technique to improve the recombination algorithm over bit sequences.

The dynamic nature of the population’s set of alleles makes maintaining a well-ordered condition a challenging problem. We propose, however, that the set of alleles does not need to be well-ordered, rather only the alleles within a block need to be well-ordered. For example, assume we are attempting to recombine the differing alleles within a block. If we analyze the first and last alleles of the block and determine that there are no recombination events between them, then we can simply copy the state of a parent sequence to the child sequence. Thus, eliminating the need to perform the classification process for this block of alleles.

We may take this a step further by requiring that every block of *W* bits reflects the same genetic structure. That is, we add the requirement that *f* assign alleles to free bits at specific positions within a block, rather than the next available. This can be achieved by considering that a theoretic upper bound, *L*, for the number of bases in a genetic sequence can be computed from the simulation parameters. The range [0,*L*) therefore represents the unit-length of each sequence. Uniformly mapping the range [0,*L*) onto the range [0,*W*) allows a bit *i* of a block to effectively represent the contiguous region $[i*\frac {L}{W}, (i+1)*\frac {L}{W})$ of the genetic sequence. In effect, each bit implicitly provides additional information about the relative genetic position of the corresponding allele.

As an example, consider a genetic sequence of length *L*=256 and a block width *W*=8. Each bit of a block represents a contiguous $\frac {L}{W}=32$ base sub-sequence. Assume a new mutation occurs at base 100 of the genetic sequence. With the unit-length requirement *f* would map 100 to bit position 3 as it represents [96,128). If there is an existing block with a free bit position 3, then the new mutation can replace the allele currently occupying that space as we described earlier. Otherwise, a new block of alleles is necessary.

By adding the unit-length requirement we gain the ability to map the set of parental regions to a block. That is, consider the set of maternal segments. We can construct a bit block where a set bit indicates an overlap between the maternal segment and the contiguous region represented by the bit position. By doing so we are able to effectively create a bit mask which can be used to select only those bit positions that potentially represent an allele residing within a maternal region.

In Algorithm 1, we construct a bit mask, *m*, which we then apply to the bit block resulting from the symmetric difference of the parental sequence blocks. Basically, *m* is used to select specific bit positions. The *buildMask* function is assumed to construct a bit mask from the set of parental regions, *R*, relative to the allele indexing function *f*. In the general case, we *buildMask* may simply return a bit block of all ones, and all bit positions resulting from the symmetric difference are selected. By changing *f* to use unit-length requirement, we can assume that the *buildMask* will react accordingly and provide the desired bit mask as described above. The remainder of the recombination algorithm remains unaffected by the change of *f*.

We present the unit-length requirement as an example of how to further utilize the ordering of alleles, rather than as a perfect solution. Block ordering alleles can aid in reducing some overheads of a recombination algorithm, though the simulation scenarios where the additional unit-length requirement is beneficial may be limited. For example, if two alleles map to the same bit position, then they cannot be represented within the same block. In other words, a unique block is necessary for each of these alleles. Thus, the addition of this requirement would likely require more memory than is theoretically necessary. As a result, we do not utilize this requirement in our experimentation.

## Results

We have performed a series of benchmarking tests to compare the scalability of our proposed approach with the approaches provided by other simulation frameworks. FWDPP [6] has adopted a designed based upon representing a genetic sequence as an ordered list of keys. SimuPOP [5] provides the ability to represent a genetic sequence as either a fixed-length binary string or as an ordered list of keys. Although these frameworks are vastly different in terms of capabilities, here we narrow our focus to comparing the memory utilization and computational runtime for common simulation scenarios. To validate our simulator we compare the average pairwise difference of populations produced by our simulator with those produced by MS [9]. All simulations are performed on a workstation computer with a 6-core Intel Xeon 3.5GHz processor, 32GB of RAM, and running 64-bit Fedora Linux version 20. Finally, all simulators utilized the Mersenne Twister random number generation algorithm (MT19937).

### Simulation scenarios

In this work, we are mainly interested in assessing the memory utilization and computational load performed in FTPGS. To do this we consider a basic evolutionary scenario where a population is evolved following a neutral mutation model with recombination. In this scenario a diploid population is evolved over a number of generations. Individuals from a parental generation are randomly selected and paired to produce to a child in the next generation. The genetic sequences of each parent are recombined and a random daughter sequence from each parent is selected to be passed along to the child. Finally, mutations are randomly introduced to the child generation.

From a functional perspective, this scenario entails the basic steps performed in all FTPGS. The simplicity of this scenario should limit sources of computational overhead to only those which result from the different sequence representations. We use this scenario to set a baseline computational runtime for each simulator.

To further evaluate the computational impact of our sequence representation, we perform an additional computational step of computing a fitness value for each individual. Although computed, the fitness value is not used in the simulation process. In effect, the evolutionary scenario remains a neutral mutation model with recombination, while demonstrating the cost of performing a more complex model. Thus, it is intended to illustrate the impact of performing a simple operation on each sequence in each generation, and how by changing the representation of a sequence can have a significant impact on performance.

While each simulation framework considered here is highly configurable, we restrict ourselves to testing these scenarios with two scales for the number of mutation events per generation, *θ*. As we are considering a diploid population, *θ* is given by 4*N*_*e*_ *μ* where *N*_*e*_ is the effective population size, and *μ* is the mutation rate per sequence. If the effective population size is considered to be constant, then changing *θ* is effectively equivalent to changing the length of a genetic sequence being simulated. A full listing of the configuration parameters by scale is provided in Table 1.

**Table 1:** Simulation scenario configuration parameters

Parameter	Symbol (equation)	Scale 1	Scale 2
Generation	*T*	100,000	100,000
Population Size	*N*=*N*_*e*_	10,000	10,000
Chromosome Length	*L*	1,000,000	10,000,000
Mutation Rate per base	*μ*_*b*_	10^−8^	10^−8^
Mutation Rate per sequence	*μ*(=*μ*_*b*_ *L*)	10^−2^	10^−1^
Recombination Rate per base	*ρ*_*b*_	10^−8^	10^−8^
Recombination Rate per sequence	*ρ*=(*ρ*_*b*_ *L*)	10^−2^	10^−1^
Mutation Events per generation	*θ*(=4*N*_*e*_ *μ*)	400	4,000
Recombination Events per generation	*P*(=4*N*_*e*_ *ρ*)	400	4,000

For simplicity, we assume a simple directional bi-allelic SNP mutation model, where the mutation rate per base is a constant rate of *μ*_*b*_=10^−8^. This assumption allows us to utilize simuPOP’s [5] *binary* module to represent a sequence, in addition to their *mutant* module. For reasons we will present shortly, we limited our experimentation with simuPOP [5] to the base evolutionary scenario, with the smaller scale configuration.

### Memory scaling

There are three genetic sequence representations being considered in this work: a fixed-length string, an ordered list of keys, and our hybrid approach. SimuPOP [5] offers the user the ability to select either a fixed-length string, or an ordered list of keys. FWDPP [6] provides an ordered list of keys representation. Finally, our Clotho library offers the hybrid approach. Our analysis of the memory utilization focuses on the idealized amount of memory necessary to represent a single generation of a population given a sequence representation. By idealized we mean that we ignore additional memory which may vary based upon implementation details, or padding introduced to maintain memory alignment.

As described above, the fixed-length interpretation of our representation relies upon the number of alleles in the population, *M*. Therefore, our representation requires a maximum of 2*N* *M* bits to represent a generation. Because we assume bi-allelic sites, *M* may be less than or equal to the length of a chromosome *L*. In the worst case then our representation will use equivalent memory to a fixed-length string. However, we represent only variable alleles within the population. In these scenarios, the variable alleles are equivalent to segregation sites under a neutral mutation model. The expectation for the total number of segregation sites in a population was described in [10], and is given by Eq. 1.

(1) $$  M = E(S) = 4N\mu \sum_{i=1}^{2N-1} \frac{1}{i}  $$

Our experimental results indicate that in the last 10,000 generations of our simulation the average number of alleles in a population to be *M*=4,173 when *μ*=0.01, and *M*=42,088 when *μ*=0.1 over the final 10,000 generations. These are within 1.2 *%* of the theoretically expected values. In both cases, *M* is significantly less than the expected sequence lengths of *L*=10^6^ and *L*=10^7^, respectively.

The ordered list of keys approach utilizes a dynamic length structure allowing for each sequence to use a minimum amount of memory. A key used to represent a mutant site is represented by a constant *K* bits. If there are *l* mutant sites within a sequence, then *lK* bits are necessary to represent the sequence.

**Fig. 2:**
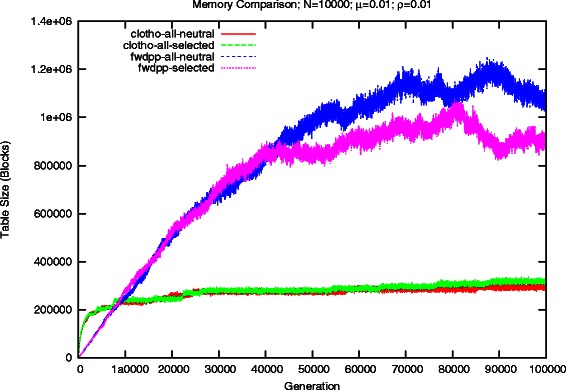
Comparison of the number of blocks need to represent a population of 10,000 individuals and a mutation rate of 0.01. Each block consists of 64 bits

**Fig. 3:**
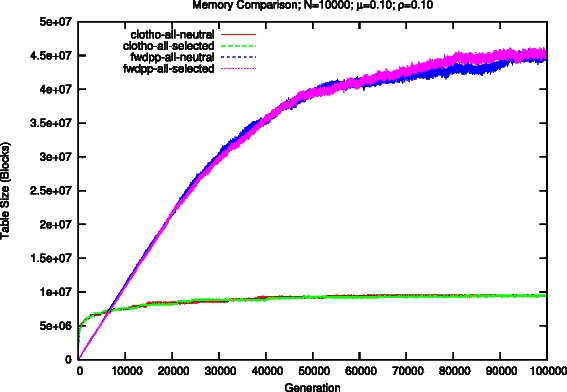
Comparison of the number of blocks need to represent a population of 10,000 individuals and a mutation rate of 0.1. Each block consists of 64 bits

Figures 2 and 3 show the total number of blocks, or keys, necessary to represent the population’s sequences for both Clotho and FWDPP [6]. The number of bits per block and key are equal to *W*=64. The early growth of *M* results in a rapid growth in the amount of memory requirements of the bit sequence representation used in Clotho. However, its growth stabilizes quickly and it grows much slower in later generations of the simulation. Conversely, the ordered list of keys used by FWDPP [6] grows relative to the number of alleles per sequence *L*. After roughly 8000 generations, the total number of blocks necessary to represent all ordered list sequences of a population begin to exceed that of population represented by bit sequences.

Although we have described a population as having 2*N* sequences, both Clotho and FWDPP [6] make use of the observation that there are likely to be duplicate sequences within the population. The idea being that if a parental sequence does not undergo mutation or recombination before being passed along to the next generation, then it is exactly the same between generations. It is therefore unnecessary to physically replicate the sequence. Simulated individuals maintain references to sequences, rather than unique copies of sequences, and a dynamic set of unique sequences is maintained. This allows Clotho to represent the sequence space in roughly 2.4 MB and 75 MB of memory when *μ*=0.01 and *μ*=0.1, respectively. Similarly, FWDPP [6] uses about 7.2 MB and 350 MB. Unfortunately, simuPOP [5] attempts to uniquely represent all 2*N* sequences of the population.

The mutant representation provided by simuPOP [5] is conceptually similar to that of FWDPP [6], although with two key differences. First, simuPOP [5] groups both a genetic position and a state to form a slightly larger *key* than that of FWDPP [6]. Second, fixed alleles are not removed from individual sequences. Consequently, the memory require per generation continually grows as more mutant alleles become fixed in the population. Combining these with the previously mentioned unique copies of each sequence, the final generation used roughly 185 MB of memory when *L*=10^6^.

We also tested the binary representation provided by simuPOP [5]. As expected, this fixed-length string representation required the most memory. For a sequence length of *L*=10^6^ simuPOP [5] required roughly 2.3 GB per generation. This is about 990x more memory than Clotho, and 320x more than FWDPP [6]. A single generation with *L*=10^7^ would require 23 GB. Unfortunately, it is often necessary to represent both a parent and child generation in memory. Therefore, we were unable to test the *L*=10^7^ scale with simuPOP because our test machine had insufficient memory.

### Runtime scaling

The design of Clotho offers several advantages which significantly reduce the computational load per generation. Perhaps the most advantageous aspect of this design is the improved data locality. Locality of reference [11], often referred to as data locality, is the concept that data elements which are close to one another tend to be operated upon together. Modern computing systems rely heavily upon this principle to enhance performance. Many of the actions taken by the system are effectively hidden from the perspective of software design. However, the implementation of software can limit the scope of possible enhancements.

As an indirect data structure, our representation only retains enough information to determine the existence of an allele in the sequence. All other information about the allele is stored in a separate data structure elsewhere in memory. It is therefore necessary to access multiple areas of memory. Generally, this is not efficient, especially when the areas are distant from one another. Fortunately, modern systems are designed with cache memory which allow distant memory locations to temporarily be moved closer together. However, cache memory space is significantly smaller than main system memory, and therefore limits the amount of data which can be relocated at a time.

The compactness of the binary sequence aids in freeing up more space for use by other objects. In effect, the system is able to utilize the additional free space, specifically free cache space, to temporarily move more of the sequence data closer to its corresponding allelic information. In other words, the indirect data structure requires additional computational work to determine allelic information. However, by having more free space available, the system is able to mitigate the cost of this work.

One may expect then that the data structures used in FWDPP [6] and simuPOP [5] would share in the benefits of cache memory, although with possibly less efficiency as they use more memory. This is generally true, however their respective implementations work against the system’s ability to utilize cache memory effectively. At a high level, both rely upon logically ordering elements in a sequence. Basically, each sequence is sorted such that the alleles are ordered based upon their genetic position, however this does not mean that each allele, or key, occurs in this order in memory. Unfortunately, the system generally relies upon the spatial locality of elements, that is their order in memory, to effectively fill the cache. While simuPOP [5] is better suited for cache efficiency, their increased sequence lengths works against them in both the mutant and binary representations.

**Fig. 4:**
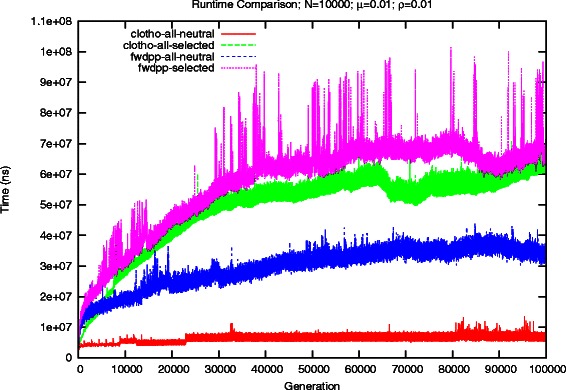
Comparison of runtime for simulated scenarios for a population of 10,000 individuals and mutation rate of 0.01

For each of the simulators and test scenarios we recorded the computational runtime time necessary to construct each generation of a population. Figure 4 shows the runtime per generation with *ρ*=*μ*=0.01 for Clotho and FWDPP [6]. In both evolutionary scenarios, Clotho offers a noticeable performance advantage. In the first scenario, Clotho is able to complete in a little over 10.1 minutes (0.006 s/generation), and FWDPP finishes in just under 49.5 minutes (0.030 s/generation). However, performing the additional step of computing a fitness value Clotho slows down significantly. While FWDPP [6] also slows, the impact of performing the additional fitness computation is much less. It was able to complete in about 95 minutes (0.057 s/generation), whereas Clotho required roughly 80 minutes (0.048 s/generation). We would expect that FWDPP [6] would end up being faster than Clotho if more generations were performed.

**Fig. 5:**
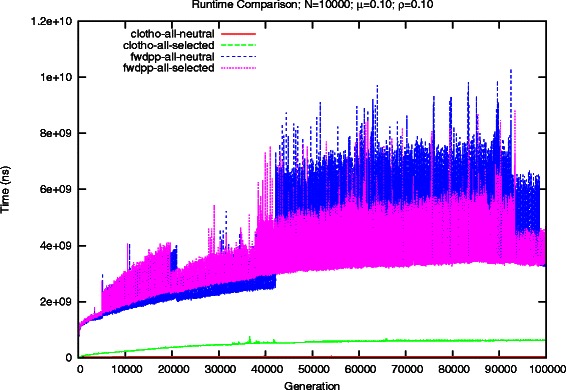
Comparison of runtime for simulated scenarios for a population of 10,000 individuals and mutation rate of 0.1

The results of the increased scale are shown in Fig. 5. In the base scenario, Clotho provides on average a 46.5x speedup per generation, reducing a 62.96 hour runtime down to 1.34 hours. A 10-fold increase in the mutation rate results in about a 76x increase in runtime for FWDPP [6], compared to an 8x increase with Clotho. Performing the additional fitness computation step expectantly increased the runtime for each simulator. Despite the additional overhead resulting from the binary representation, Clotho noticeably reduces the runtime of a simulation, completing in 13.7 hours compared to 83.8 hours.

**Fig. 6:**
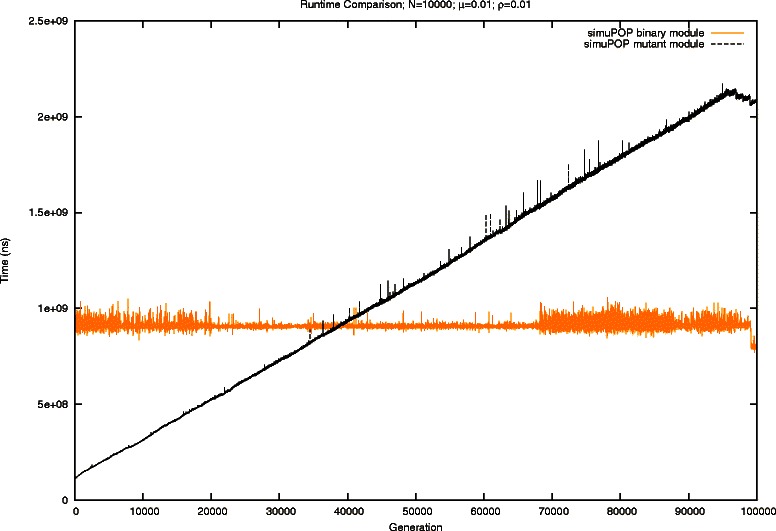
Comparison of runtime for simuPOP [5] for a population of 10,000 individuals and mutation rate of 0.01. The black line represents the mutant module, and the binary module is the orange line

The results of performing the evolutionary scenario at the smallest scale using simuPOP [5] are shown in Fig. 6. The binary module requires a relatively constant amount of time to generate each generation. This is expected given the simulation scenario. The linearly increasing runtime for the mutant module is also expected. It results from the increasing number of fixed mutant alleles. In terms of total runtime, simuPOP [5] needed 25.3 hours when using the binary module, and 31.89 hours with the mutant module. Clotho offers a speedup of over 145x and 185x, respectively.

### Other scales

We have also studied how the Clotho approach scales with larger population sizes, *N*, and mutation rates, *μ*. We focused our efforts on only the base evolutionary scenario as this would represent a lower bound for runtime. Tables 2, 3 and 4 show the results.

**Table 2:** Average number of alleles (*M*) in a population over *T*=100,000

1-3	10,000	20,000
0.01	3,971	8,011
0.1	39,803	80,379
1.0	396,816	804,105

Rows are mutation rate (*μ*). Columns are Population Size (*N*)

**Table 3:** Average Total Size (MB) of F as bit sequences over *T*=100,000 generations

1-3	10,000	20,000
0.01	2	8
0.1	70	260
1.0	1,100	4,400

Rows are mutation rate (*μ*). Columns are Population Size (*N*)

**Table 4:** Total Runtime (hours) of Clotho over *T*=100,000

1-3	10,000	20,000
0.01	0.2	0.4
0.1	1.3	5.1
1.0	41.8	167.4

Rows are mutation rate (*μ*). Columns are Population Size (*N*)

As expected, the number of alleles in the population, *M*, grows proportionally with the increase in *N* and *μ*, respectively. The memory required to represent the sequence space also increases accordingly. Interestingly, increasing the mutation rate from *μ*=0.1 to *μ*=1.0 results in roughly a 32x increase in runtime, whereas the increase from *μ*=0.01 to *μ*=0.1 was roughly an 8x increase.

### Validation

The base evolutionary scenario considered in this work generates a population under the a neutral mutation model with recombination. MS [9] is an efficient tool which is also capable of generating populations under this process. To validate our simulator we compared the population produced in the final generation of our simulation scenarios with a population produced by MS [9] using the appropriate configuration parameters. Specifically, we compare the average pairwise difference, between a sample of 200 sequences randomly selected from each population.

Under the neutral model of evolution we would expect that the average pairwise difference would be equal to the number of segregation sites. The number of segregation sites is expected to be *θ*. We consider recombination in our simulation. Therefore, we expect there to be some deviation from the theoretical prediction. The results are shown in Table 5.

**Table 5:** The average pairwise difference for a random sample of 200 sequences from a population of 20,000 with *θ*=*ρ*=400 and *L*=100000

1-3	Observed	Expected
MS	398	400
Clotho	405	400

## Discussion

In this manuscript, we have described a method of representing a genetic sequence of alleles as a bit sequence for FTPGS. We have shown that this representation is effective in improving the scalability of these simulations by comparing our representation with a more commonly utilized ordered list of keys. In this section we will present some scenarios where this representation may not be well suited. Also, while the ordered list of keys representation is common, we discuss some related works that provide alternate genetic sequence representations. Finally, we will offer some future directions we intend to pursue.

### Limitations

As we alluded to earlier, our utilization of a binary sequence to represent a subset of alleles has some disadvantages. Recall that each sequence is an implicit data structure. That is, the relative position of set bits is used to identify the alleles are in each sequence. An algorithm that requires information specific to an allele has to perform a translation step to first identify a set bit then map its relative position to the allele. It is beyond the scope of this work to discuss the different techniques of identifying set bits. It suffices to say that each has an unavoidable computational cost, and can become significant in a large scale simulation scenario. In effect, we have traded a level of computational performance in an attempt to save memory. Conversely, there are simulation scenarios for which this representation will increase memory requirements.

In general, the worst case scenario for our binary representation is one in which the set of alleles for each generation grows, but the number of alleles per sequence remains comparatively small or fixed. If new alleles are introduced to a child generation more frequently than alleles are fixed or lost in the parent generation, then our binary representation suffers. In computational terms, our representation suffers from becoming increasingly sparse.

Our approach does attempt to combat the issue of increasing sparsity. Recall that we re-use bit indices associated with non-variable alleles in the parent generation for alleles which are introduced in child generation. In this way, we are able to maintain a minimal set of alleles for each generation. While maintaining a minimal set of alleles is ideal, in some simulation scenarios, such as those which consider an infinite allele mutation model [12], the increasing number of alleles may be unavoidable. In these scenarios, utilizing an ordered list of keys would be more advantageous.

Finally, our reliance on an allele indexing function to map between bit positions and alleles may be a bottleneck in some algorithms. Many traditional implementations of evolutionary models rely upon the input genetic sequences being well ordered. Generally our design does not have this guarantee. Therefore, additional work is necessary if a traditional implementation must be used. For example, although being generally inefficient, our sequences can always be translated into an equivalent ordered sequence. However, as we continue development of this work, we intend to provide implementations of evolutionary models which do not assume ordered sequences.

#### Relate works

Recent works by [8, 13, 14] have suggested that representing a genetic sequence as a sequence of haplotype units provides substantial benefit in FTPGS. Haplotype units are segments of a genetic region where all alleles within the segment are inherited together. This allows for more efficient construction of child sequences. It also improves fitness computation as the haplotypes phenotypic contribution of a region can be pre-computed [13]. Furthermore, the ancestral information of a haplotype region can be maintained, allowing for the full ancestral history of a sequence to be reconstructed [8].

In [8], they utilize bit arrays when representing neutral sequences that are constructed from their BEG algorithm. They also perform bitwise operations in the algorithms for inheriting mutations in a haplotype unit in a parent sequence. While this is primarily what we have proposed in this work, it does differ in that the construction of sequences is performed at a point when all mutations are known. As a result, they are able to order all mutations without relying upon the definition of an allele-indexing function.

Our reliance on the definition of an allele indexing function is a key feature of our design. It allows us to efficiently maintain the dynamic set of alleles. While we have framed this manuscript with a single-base allele as our basic element, our design is flexible and not limited to such alleles. We believe that our design enables the creation of simulators which are based upon haplotype units without difficulty. We intend to explore this in the near future.

### Future directions

We plan to expand the genetic models available in Clotho. Currently, we only provide some basic models of recombination, mutation, and fitness. We intend to incorporate models for quantitative traits, as well as functionality to retain ancestral information. Certainly, we would like to explore the use of haplotype units as the basic element in our design.

From a computational perspective, we intend to explore the use of parallelism to further reduce the runtime of FTPGS. In many respects, FTPGS are embarrassingly parallel problems. The majority of the simulation can be expressed as a series of independent tasks. This task level parallelism can be exploited through the use of multithreading capabilities of a standard workstation. Having said this, our bit sequence representation opens the door for additional parallelism.

We showed earlier that Boolean Algebra operations being applied to blocks of alleles. Since the blocks of alleles of a sequence are independent, it follows then that the task of applying a Boolean Algebra operation to an entire sequence is an embarrassingly parallel problem. While the multithreading available on a CPU may also be utilized to improve these tasks, a GPGPU was designed to handle parallel problems of this nature. We plan to explore the use of a GPGPU to perform FTPGS.

## Conclusion

Our design of Clotho is a general way for improving the scalability of FTPGS on a basic desktop with a single CPU and low amounts of memory. We have shown that by representing genetic sequences as bit sequences we are able reduce FTPGS memory requirements by roughly 4x. Furthermore, the compact representation allows for the use of Boolean Algebraic operations in many algorithms. This aids in improving the runtime of larger scale simulation scenarios, with some running as much as 46x faster than an equivalent ordered list of keys representation. Some algorithms are negatively impacted by the implicit data representation incurring significant amounts of computational overhead which results from bit walking. However, the additional overhead is often acceptable as larger scales are still computable in significantly reduced times. We intend to continue to expand the capabilities of Clotho by incorporating more genetic models, and the ability to utilize parallel computing hardware such as a GPGPU.

## Availability

Clotho is implemented as an open source C++ template library. The source code and example simulators constructed from this library are available as a GitHub project at http://github.com/putnampp/clotho.
